# Estimation of the Structure of Hydrophobic Surfaces Using the Cassie–Baxter Equation

**DOI:** 10.3390/ma17174322

**Published:** 2024-08-31

**Authors:** Oleksiy Myronyuk, Egidijus Vanagas, Aleksej M. Rodin, Miroslaw Wesolowski

**Affiliations:** 1 Department of Chemical Technology of Composite Materials, Chemical Technology Faculty, Igor Sikorsky Kyiv Polytechnic Institute, Beresteiskyi Avenue 37, 03056 Kyiv, Ukraine; 2 Coherent Optics Laboratory, Department of Fundamental Research, Center for Physical Sciences and Technology, Sauletekio Avenue 3, 10257 Vilnius, Lithuania; egisvan@gmail.com; 3 Solid State Laser Laboratory, Department of Laser Technologies, Center for Physical Sciences and Technology, Savanoriu Avenue 231, 02300 Vilnius, Lithuania; aleksej.rodin@ftmc.lt; 4 Department of Structural Mechanics, Faculty of Civil Engineering, Environmental and Geodetic Sciences, Koszalin University of Technology, Sniadeckich Street 2, 75-453 Koszalin, Poland

**Keywords:** contact angle, wetting, superhydrophobic, polymer, Cassie–Baxter equation

## Abstract

The effect of extreme water repellency, called the lotus effect, is caused by the formation of a Cassie–Baxter state in which only a small portion of the wetting liquid droplet is in contact with the surface. The rest of the bottom of the droplet is in contact with air pockets. Instrumental methods are often used to determine the textural features that cause this effect—scanning electron and atomic force microscopies, profilometry, etc. However, this result provides only an accurate texture model, not the actual information about the part of the surface that is wetted by the liquid. Here, we show a practical method for estimating the surface fraction of texture that has contact with liquid in a Cassie–Baxter wetting state. The method is performed using a set of ethanol–water mixtures to determine the contact angle of the textured and chemically equivalent flat surfaces of AlSI 304 steel, 7500 aluminum, and siloxane elastomer. We showed that the system of Cassie–Baxter equations can be solved graphically by the wetting diagrams introduced in this paper, returning a value for the texture surface fraction in contact with a liquid. We anticipate that the demonstrated method will be useful for a direct evaluation of the ability of textures to repel liquids, particularly superhydrophobic and superoleophobic materials, slippery liquid-infused porous surfaces, etc.

## 1. Introduction

The enhanced ability of textured surfaces to repel liquids, inspired by natural examples such as leaves and flowers of plants (rose, lotus) and insect organs (lint on the body of bees and mosquitoes), has been extensively studied in recent years [1,2,3]. Significant attention from research groups is focused on reproducing the necessary textures based on new materials that allow them to increase the intrinsic non-polarity of the surface [4]. Various methods for obtaining such textures are also being considered, which can be roughly categorized into two types—additive and extractive. The former includes coating surfaces by a variety of methods [5,6] (solution deposition, vapor deposition). The latter is based on the removal of part of the material surface (electrochemical etching [7] and laser treatment [8]), which results in the formation of texture.

At the moment, despite the extensive amount of research, the problems formulated in [9], which prevent the evolution of experimental findings into industrially suitable solutions, remain relevant. The main problems are the insufficient scalability of the solutions, the high cost of the texturing process, and the insufficient adaptability of the theoretical framework for evaluating the relationship between surface texture features and water repellency. In particular, this concerns the practical applicability of the basic equations of Cassie and Wenzel, as shown in our previous study [10].

The Cassie–Baxter Equation (1) contains the parameters $f_{1}$ and $f_{2}$, which characterize the fractions of the liquid–solid and liquid–air contact surface from the air cavities of the texture:

(1) $$cos\theta_{\mathrm{app}}=f_{1}cos\theta_{1}+f_{2}cos\theta_{2}$$

where $cos\theta_{\mathrm{app}}\;$ is the contact angle of a composite two-phase surface: $f_{1}$ and $cos\theta_{1}$ are the fraction of the surface and the contact angle of phase 1, and $f_{2}$ and $cos\theta_{2}$ are the fraction of the surface and the contact angle of phase 2.

And it is intuitive that the sum of these fractions should sum to one; however, as is shown in [11], this assumption works only for those textures that are composed of irregularities with rectangular cross-sections of the tips of the asperities. In that case, the surface of the droplet is in contact with essentially only part of the flat surface, and the other part extends over air pockets of such texture. However, by changing the geometry of the asperities, the value of $f_{1}$ increases relative to the case with rectangular cross-sections, and the sum $f_{1}$ + $f_{2}$ is not equal to one. This position can also be supported by the Wenzel wetting state in Equation (2), which is essentially a reduction in the Cassie–Baxter equation and an expression of the state in which air pockets do not exist and the contact surface is no longer heterogeneous:

(2) $$cos\theta_{app}=r\times cos\theta_{0}$$

where $cos\theta_{\mathrm{app}}$ is the wetting angle of the textured surface, $cos\theta_{0}$ is the wetting angle of the corresponding flat surface, and *r* is the roughness parameter, which is the ratio of the total surface area to the area of its projection.

The minimum value of the parameter *r* represented in this equation is one and increases as the surface roughness increases.

From Equation (1), it follows that the two main factors that allow the repulsion of liquids to be achieved are texture parameters ($f_{1}$ and $f_{2}$) and the natural energy of the surface, which is characterized by the parameter $cos\theta_{1}$.

Obviously, knowledge of the geometric parameters of the Cassie state ($f_{1}$ and $f_{2}$) is one of the credible ways of practically assessing the effectiveness of liquid-repulsive texture. However, the existing methods of their determination, based on information about the surface topography—atomic force microscopy, scanning electron microscopy [12,13]—are rather resource- and time-consuming and, in addition, indirect. In particular, the determination is carried out on individual surfaces and not at the moment of wetting contact formation. As it is shown in [14], the deviation between the calculated values of the contact angle using the topography data and the measured values may reach up to 15–25°.

The objective of this paper is to formulate a practical method for estimating the parameters of the Cassie equation, $f_{1}$ and $f_{2}$, which can be used for the rapid determination of a texture’s ability to repel liquids and for comparing the quality of hydrophobic textured materials.

For this purpose, hydrophobic textures on the surface of anodized aluminum and steel with different but fixed and highly reproducible structural parameters were obtained by femtosecond laser ablation and subsequent modification with octyltriethoxysilane. The contact angle values were obtained for a series of different surface tension probe liquids and mixtures of ethanol with water, which ensured a decrease in the surface tension increments in the range of 30–72 mN/m. Furthermore, wetting diagrams were constructed on the basis of static contact angle measurements. Using a graph, the values of the Cassie equation parameters were obtained and compared with the parameters derived from the geometrical characteristics of the structure. The use of this characterization technique was additionally demonstrated on the surfaces of a silicone polymer textured by the templating.

## 2. Materials and Methods

The application of this method is illustrated on surfaces obtained by femtosecond laser treatment of 7500-grade anodized aluminum and AlSI 304 steel (Outokumpu, Torino, Finland). The texturing was performed using an air-cooled “Carbide” laser (Light Conversion, Vilnius, Lithuania), as described in detail in [10]. The methods of preparation and surface treatment are also described in detail in our previous works [10,15], respectively.

In addition to these samples, we fabricated a negative cast based on the additional cured system that consisted of 1000s St vinyl-terminated polydimethylsiloxane (PDMS) DMS-V31 (100 parts) and methylhydrosiloxane-dimethylsiloxane copolymer HMS-301 (3 parts). The Karstedt platinum complex catalyst was used to cure the system SIP6830.3 (200 ppm). All materials were obtained from Gelest Inc., Morrisville, PA, USA. After the mixing of the substances, the system was poured onto the surface of the pre-cleaned textured metal template and placed under the vacuum for 120 s to remove entrapped air bubbles. The system was brought to ambient pressure and temperature and left for 3 days. The polydimethylsiloxane (PDMS) template was removed from the cast by carefully pulling it off.

The contact angle of the PDMS cast surface was 145°, which was caused by the intrinsic low surface energy of this polymer, 18–21 mN/m [16] and, therefore, it is often used to produce water-repellent surfaces [17,18,19]. The anodized aluminum and steel samples were treated with otyltrimetoxysilane (OCTEO) (CAS 3069-40-7) to place them at a similar level of hydrophobicity required for the Cassie wetting anomaly occurrence. 

The contact angle value was determined with the use of an optical microscope and a digital camera, following the technique described in [20]. The test droplets were applied at five different predetermined points on the sample surface. After each measurement, the samples were dried for 60 s at 60 °C. Considering that the illustrated method requires an increased amount of probe liquids and a limited quantity of existing individual liquids with suitable surface tension ranges, mixtures of ethanol–water were used. The surface tension of these liquids was calculated using the method from [21]. This liquid pair was selected because of the following practical reasons: existing and previously reported calculations and the availability of the base liquids. Furthermore, the surface tension of both liquids, water (72.5 mN/m) and ethanol (22.4 mN/m) covered the range where the Cassie–Wenzel wetting transition occurred. 

The geometry and morphology of the textures were characterized by scanning electron microscopy (SEM) (MIRA, Tescan, Brno, Czech Republic) with an acceleration voltage of 20 kV and a secondary electrons (SEs) detector. Prior to the characterization, to avoid charging, the surfaces were coated with a 10 nm platinum–palladium layer via magnetron sputtering.

## 3. Results

### 3.1. Surface Textures

**Figure 1:**
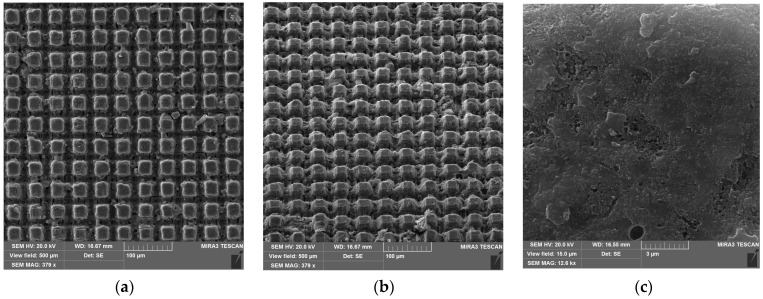
Surface structure of Al-46-16 sample (**a**) top view; (**b**) side view; and (**c**) surface of the asperity.

**Figure 2:**
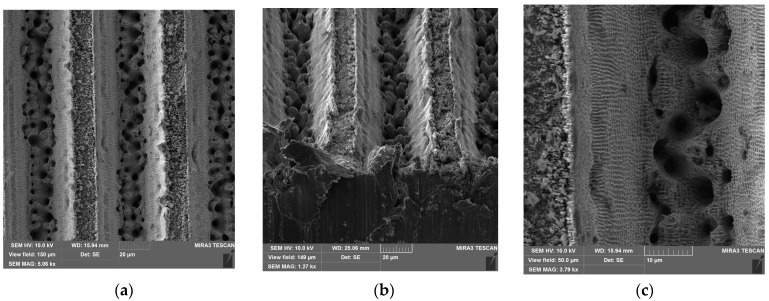
Surface structure of St-60-45 sample (**a**) top view; (**b**) side view; and (**c**) groove border.

**Figure 3:**
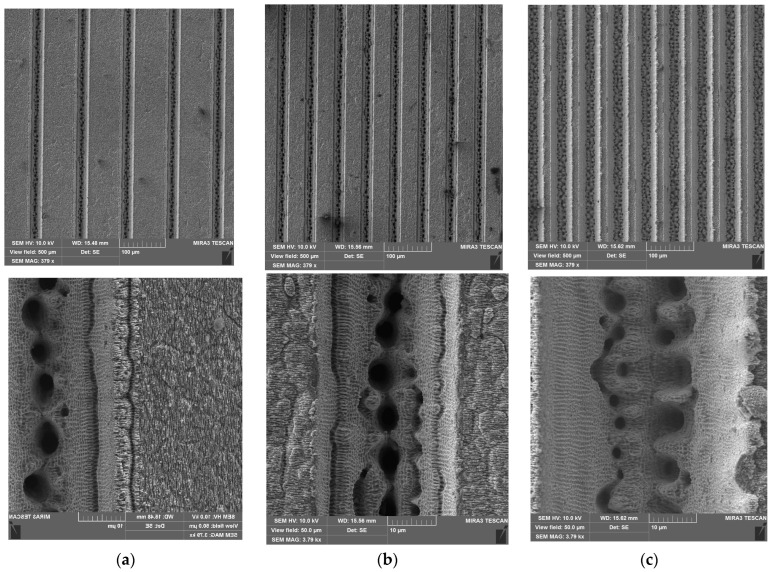
Surface structure and asperities/groove borders of samples (**a**) St-100-30-L; (**b**) St-60-30-L; and (**c**) St-60-45-L.

Textures with different geometric parameters obtained on metal surfaces by femtosecond laser ablation were used as model surfaces (Table 1). The samples on the AlSI 304 steel surface were named “St”, and on the anodized aluminum—“Al”. The first number in a sample name is the structure period in µm, and the second is the groove width in µm. The steel samples with a laser-induced periodic surface structure (LIPSS) on top of the asperities are additionally marked “L”. Further details about the texture configuration may be understood from the following SEM photos (Figure 1, Figure 2 and Figure 3).

The surface of the sample Al-46-16 (Figure 1) is composed of cubic asperities with a height of 16 µm and the same spacing distance between them. Note that the surface of the alumina on the top of the asperities is relatively smooth and even, without an additional level of texture. The fraction of the top of the asperities area is 0.126 of the total projection area of the sample.

The steel sample St-60-45’s texture (Figure 2) is composed of parallel grooves with a spacing on the top of 45 µm. The asperities’ top structure, however, is not even, as in the case of the previous sample, but contains lifted borders (probably caused by the deposition of the metal melt) and the inner texture of a submicron scale, probably formed due to the debris of the removed metal re-deposition and the following oxidation. It has to be taken into account that such borders may form additional capillaries if they are high enough to keep the droplet of wetting liquid from touching the surface of the crystalline layer.

The surface patterns of samples St-60-45-L, St-60-30-L, and St-100-30-L are represented by parallel groves with different widths and periods (Figure 3). In this sequence, the fraction of the surface area of the asperities increases. The surface of the asperities is covered by an LIPSS-like texture, which was formed prior to the formation of the grooves. It is noteworthy that the borders of the grooves of these samples are significantly lower than in the case of sample St-60-45, so the additional capillary formed by the residue metal is lower.

Thus, the formed structures differ both in type (linear and lattice) and in the presence of additional microstructures on the surfaces of the elevations. It is known that immediately after the treatment of these surfaces with a laser, they are hydrophilic because the steel, Al materials, and their outer oxides have a significant share in the acid-base components of the surface energy. Therefore, their water contact angles are 51° for 304 steel, 70 for aluminum [22], and 10° for anodized aluminum [23], which means that the Cassie state is not reached on such hydrophilic surfaces. The hydrophobization of oxides is possible in various ways; for example, even with simple exposure to air, a wetting transition is observed as, due to high adsorption activity, such surfaces attract hydrocarbon contaminants from dry air [24,25]. However, more stable hydrophobicity is achieved using chemical treatments, namely reactions with silanes, their fluorinated forms, carboxylic acids, etc. [26]. In this work, all surfaces were treated with octyltriethoxysilane (OCTEO) after texturing.

### 3.2. Method Equation

The obtained textures after hydrophobization are water-repellent due to the formation of the Cassie state. The expected value of the contact angle with liquid, in this case, is determined by the Cassie–Baxter Equation (1), taking into account the fact that the water contact angle of air (as the second phase of the composite surface) is equal to 180°. Then, if *cosθ*_2_ is equal to −1, the resulting equation will be written as follows (3):

(3) $$cos\theta_{\mathrm{app}}=f_{1}cos\theta_{1}-f_{2}$$

In this form, the equation captures the idea of creating surfaces that repel liquids. To increase the contact angle $\theta_{\mathrm{app}}$, that is, the decrease in $cos\theta_{\mathrm{app}}$, this can be achieved as follows: a. $f_{2}$ should be increased so it is the same as the increase in the droplet air pockets’ surface fraction; b. lower the $f_{1}$ value so the wet part of the surface is less curved; and c. decrease $cos\theta_{1}$, which may be performed via surface polarity reduction through hydrophobic treatment or with intrinsically hydrophobic materials for the surface construction.

Equation (3) has two unknown values, $f_{1}$ and $f_{2}$, which can be found by the well-known method of solving a system of equations. This approach is used, for example, in the Owens–Wendt method [27,28]. For this purpose, it is necessary that these two unknowns do not change when the pair $cos\theta_{\mathrm{app}}$ and $cos\theta_{1}$ change. This would be possible using surfaces with the same geometry made of materials of different polarities or by gradually increasing the polarity of an initially hydrophobic material (e.g., by photodegradation). Another more practical way would be to change not only the polarity of the surface but the surface tension of the probe liquids. In this case, in accordance with the fact that the contact angle of a material decreases when its surface tension decreases, the following should change $cos\theta_{\mathrm{app}}$ as well. The number of probe liquids, in this case, determines the number of equations of the form (3) in the system. Systems of equations can be solved graphically.

### 3.3. Graphical Solution of Equations and Wetting Diagrams

**Figure 4:**
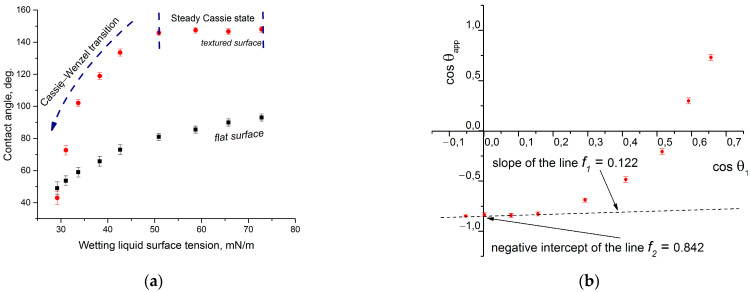
Wetting of the flat and textured anodized aluminum surfaces (treated with OCTEO) with different surface tension liquids: (**a**) the dependence between the contact angle for the flat and textured surfaces, (**b**) the wetting diagram.

Figure 4a shows the dependence of the contact angle of the texture (Al-46-16) and the flat surface of the hydrophobized anodized aluminum on the surface tension of the wetting liquid. The sample Al-46-16 has a characteristic plateau where there is no appreciable decrease in the contact angle value; i.e., the Cassie state in this range of the surface tension of the probe liquid remains stable. Lower than 52 mN/m, the wetting transition to the Wenzel state occurs. The wetting diagram Figure 4b is plotted with coordinates of the dependence (3). The graph of this dependence also contains a plateau at the beginning, the points on which can be approximated by a straight line. The intersection of this line with the Y-axis gives us the numerical value of the negative parameter $f_{2}$. For the sample Al-46-16, it is 0.842. And the slope of this straight line delivers the $f_{1}$ value (0.122).

The parameters obtained in this way are close to the geometric parameters that can be calculated by SEM image analysis (Figure 1). Assuming that the surface roughness of the upper plane of the elements of this texture is insignificant, we can assume that the surface area of the asperities is equal to its projection and is 0.126 of the total projection area of the sample. From such considerations, the parameter $f_{1}$ = 0.126 and the parameter $f_{2}$ = 1 − 0.126 = 0.874 since the protrusions can be considered conventionally rectangular. The values obtained in this way are close to those obtained in the wetting diagram analysis (Figure 4b), which confirms the validity of the assumptions made. 

**Figure 5:**
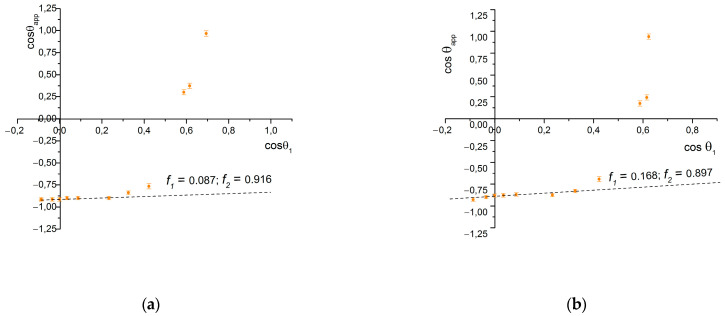

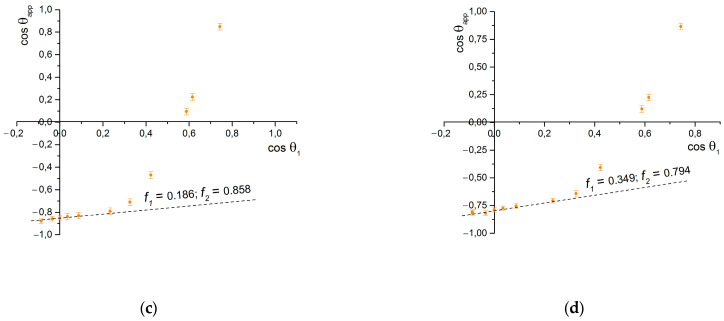
Wetting diagrams of samples: (**a**) St-60-45; (**b**) St-60-45; (**c**) St-60-30-L; and (**d**) St-100-30-L.

Wetting diagrams of samples on a steel surface (Figure 5) allow us to determine the wetting parameters of these textures, which, however, significantly diverge from those determined geometrically (Table 2). In particular, the geometric parameter $f_{1}$ is several times larger than that determined by wetting, and the geometric parameter $f_{2}$, by contrast, is lower. This can be explained by the fact that when calculating the geometric parameters, the upper surface of the asperities was assumed to be flat, without significant roughness, which turned out to be an incorrect assumption. 

The presence of additional roughness on the surface of the asperities determines the fact that the fraction of the droplet surface that directly contacts the surface decreases, and the area of contact between the lower surface of the drop and the air pocket increases. 

This is especially evident in the case of the sample St-60-45 (Figure 2), which, together with a lifted border of 1–2 µm in width, has an additional level of the top texture formed by the re-deposition of debris and the resultant oxidation. It lets the sample achieve an enhanced water-repellent property in comparison to other samples. Sample St-60-45-L and all others from the set have an additional LIPSS texture on their surfaces that, as it is known [29], provide increased water repellency through the formation of the Cassie state. The edges of the asperities of these samples are also more uniform, which does not provide the same effect as the additional structure, as in the case of sample St-60-45 (compared with St-60-45-L (Table 2)), which has an identical pattern of microgrooves. This explains the increase in the parameter $f_{1}$ by almost twofold if the LIPSS structure is present. When increasing the area of the top of asperities in a row, St-60-45-L → St-60-30-L → St-100-30-L, the parameter $f_{1}$ increases as expected, and the parameter $f_{2}$—decreases.

### 3.4. Application on Textured Polydimethylsiloxane Surface

**Figure 6:**
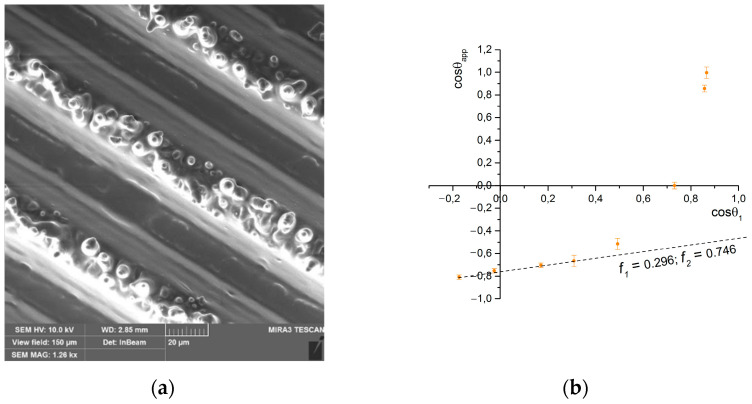
Texture (**a**) and wetting diagram (**b**) of the PDMS sample surface formed by the templating technique.

To expand the list of surface types to illustrate the performance of the method, a sample of cured polydimethylsiloxane was obtained using the sample St-60-45 as a template. The obtained cast (Figure 6a) is the negative of the structure of the original surface. As can be seen, the bottom of the siloxane cast grooves is relatively flat, but the asperities have a subtexture in the form of smooth and truncated cones. Their origin becomes clear when we take a closer look at the structure of the depressions of the original sample St-60-45 (Figure 2). Its bottom is mottled with pits—artifacts from the interaction of the focus of laser pulses with the surface of the material. A similar shape of pits on the surfaces of samples as a result of short-pulse lasers is described in [30]. The height of these pyramids is up to 5 μm, and the diameter of the base varies between 2 and 6 μm.

It is known that PDMS is a hydrophobic polymer; the wetting angle of its flat surface is 100° (which agrees with the works [31,32]), so such textures do not require additional hydrophobization. The water-wetting angle of the impression surface is 145°. Moreover, this structure has a rather high value of the parameter $f_{1}$—0.296, which follows from the corresponding wetting diagram (Figure 6b).

This can be explained by the fact that pyramids of protrusions are not effective from a geometrical point of view, and their surfaces, during contact with a drop of liquid, are wetted due to the sagging of the drop under its weight, increasing the parameter $f_{1}$. The pyramids are also movable and can change position when in contact with a drop of probe liquid. However, such geometries can find uses in other areas. For instance, the possibility to use pyramidal tips in force spectroscopy measurements exists [33] to sense spontaneous capillary bridges when the tip is placed a few nanometers -distant from the substrate surface [34], the extension of which is directly related to the hydrophilic nature of the tested material.

**Figure 7:**
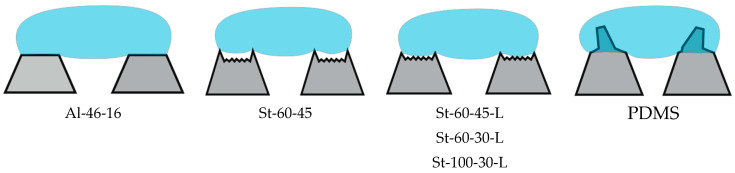
Schematic illustration of contact between the wetting liquid and the studied surfaces.

Thus, using wetting diagrams to analyze the repellent efficiency of liquids of a particular structure, we can generalize the types of asperities surfaces/liquid contacts (Figure 7) for the sample groups under consideration.

Thus, the analysis of wetting diagrams gives an idea of the distribution of the fractions of the lower surface of a droplet that is in contact with the air trapped in substrate irregularities. Based on this, the effectiveness of certain types of water-repellent structures can be evaluated. This method may be particularly useful for a deeper analysis of superhydrophobic surfaces than just the values of wetting and rolling angles, given the considerable number of published studies and results on the synthesis of such surfaces from various materials [35,36,37]. It should be noted that this method can only be used on surfaces where the Cassie state is achieved. The accuracy of the method increases if the material has a stable plateau of this state; then, the linear approximation of the experimental data is more significant.

### 3.5. Description of the Method

The aim of this subsection is to formalize the method’s procedure to make it practically useful for Cassie–Baxter parameters and the determination of textured liquid-repellent surfaces. The method is applicable only for a surface-liquid pair that exhibits a steady Cassie wetting anomaly. This fact can be seen from the existence of *the plateu*—the plane section of the dependency contact angle = f(the surface tension of probe liquid), as shown in Figure 4a of this paper.

**Figure 8:**
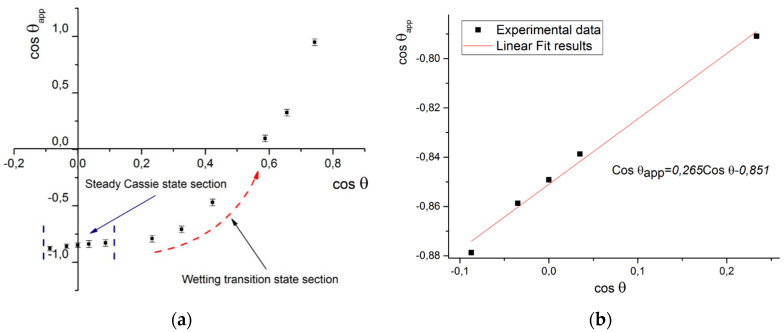
Graphical solution description: (**a**) example of the wetting diagram; (**b**) results of linear fitting of Cassie state points.

To plot the wetting diagram (the graphical representation of Equation(3)) for the set of the probe liquids, the following has to be determined: (1) the contact angle of this particular liquid and the textured surface and (2) the contact angle of a chemically identical flat surface. Then, their cosines have to be calculated, and the graphical dependence $cos\theta_{\mathrm{app}}=f\left(cos\theta_{1}\right)$, called in this work “the wetting diagram,” is drawn (Figure 8a).

It is important to identify when *the plateu* region ends. This can be determined by the simple identification of where the linear part of the curve ends and where the Cassie state is lost, and the method is not applicable for such data points. In the case of the example illustrated in Figure 8a, suitable data points are indicated as the “Steady Cassie state section”.

The next step is the linear regression fit of the selected points, which may be performed with various software (in this paper, we used OriginPro 2015 b.9.2.257), which will give the resulting equation (Figure 8b). The intercept is −0.856, and the slope is 0.265. These values correspond to the *f*_2_ and *f*_1_ Cassie–Baxter equation parameters, respectively.

This method requires a set of probe liquids with known surface tensions that fit within the plateau region of the surface under study.

## 4. Conclusions

A practical method for estimating $f_{1}$ and $f_{2}$, parameters of the Cassie equation is formulated based on the determination of static angles of the wetting of surfaces by a set of test liquids with different surface tensions. This method may be useful for rapidly determining the ability of textures to repel liquids and for comparing the quality of hydrophobic textured materials.

It is shown that during the formation of microtextures by femtosecond laser ablation on the surfaces of anodized aluminum and steel, water-repellent properties are determined, along with the configuration of micropatterns, which are also determined by the type of substructure on the surfaces of the asperities. The least effective, in terms of repelling liquids, is the flat surface of the asperities ($f_{2}$ = 0.842), which increases in the presence of an additional LIPSS texture and increases in the widths of microgrooves ($f_{2}$ = 0.897). The most effective texture is one that contains artifacts from texturing—walls at the edges of grooves ($f_{2}$ = 0.916).

The PDMS surface formed by the template method based on the surface texture obtained by femtosecond laser ablation contains artifacts in the form of truncated cones on the surface—traces from the laser pulse interaction. This additional level of texture, however, is not effective in forming stable water repellency ($f_{2}$ = 0.842).

The method illustrated in this paper can be useful for finding the actual coefficients of the Cassie–Baxter equation without using indirect methods. It is convenient because it uses relatively simple equipment for determining the wetting angle using the sitting drop method and a set of calibrated test liquids. This method is useful for the non-harmful determination of the effectiveness of liquid-repellent textures, finding the optimum surface configuration, etc.

## Figures and Tables

**Table 1:** Sample texture parameters.

Sample	Texture Period, µm	Groove Width, µm	Asperities Surface Features
St-60-45	60 ± 1.9	45 ± 1.9	Crystal
St-60-45-L	60 ± 1.9	45 ± 1.9	LIPSS
St-60-30-L	60 ± 1.9	30 ± 1.2	LIPSS
St-100-30-L	100 ± 2.1	30 ± 1.2	LIPSS
Al-46-16	46 ± 1.9	16 ± 1.0	flat

**Table 2:** Surface fraction in contact with solid ($f_{1}$) and air in pockets ($f_{2}$) calculated from SEM and measured by wetting.

Sample	Geometrical (from SEM)		Measured by Wetting	
	$f_{1}$	$f_{2}$	$f_{1}$	$f_{2}$
St-60-45	0.25 ± 0.015	0.75 ± 0.045	0.087 ± 0.006	0.916 ± 0.07
St-60-45-L	0.25 ± 0.015	0.75 ± 0.045	0.168 ± 0.008	0.897 ± 0.07
St-60-30-L	0.50 ± 0.015	0.50 ± 0.030	0.186 ± 0.008	0.858 ± 0.07
St-100-30-L	0.70 ± 0.015	0.30 ± 0.015	0.349 ± 0.009	0.794 ± 0.06
Al-46-16	0.126 ± 0.008	0.874 ± 0.043	0.122 ± 0.012	0.842 ± 0.06

## Data Availability

The original contributions presented in the study are included in the article, further inquiries can be directed to the corresponding author.
